# Power and Phase Fusion Spectrogram with Three-Dimensional Convolution and Vision Transformer for Seizure Detection

**DOI:** 10.3390/diagnostics16132012

**Published:** 2026-06-27

**Authors:** Yuyue Jiang, Zhuohan Wang, Yazhou Zhao, Weidong Zhou, Guoyang Liu

**Affiliations:** 1 School of Integrated Circuits, Shandong University, Jinan 250101, China; 2 Shenzhen Research Institute of Shandong University, Shenzhen 518000, China; 3 Tandon School of Engineering, New York University, New York, NY 10012, USA; 4 Key Laboratory of Social Computing and Cognitive Intelligence, Dalian University of Technology, Ministry of Education, Dalian 116024, China

**Keywords:** seizure detection, continuous wavelet transform, 3D convolutional neural network, vision transformer

## Abstract

**Background/Objectives:** Reliable detection of epileptic seizures using electroencephalography (EEG) is crucial for clinical diagnosis and for alleviating clinicians’ workload. However, existing studies still make insufficient use of phase information, and the synergy between local time–frequency pattern extraction and global dependency modeling remains limited. **Methods:** We propose a seizure detection framework based on the continuous wavelet transform (CWT), a three-dimensional convolutional neural network (3D-CNN), and a vision transformer (ViT). First, multichannel EEG segments are preprocessed, after which CWT is used to generate power spectrograms and phase spectrograms. These representations are then fused along the depth dimension into a unified power-phase volume and fed into a hybrid network composed of a 3D-CNN feature extractor and a single-layer ViT encoder to jointly learn local time–frequency–channel coupling patterns and higher-level global dependencies. Finally, seizure detection is completed by combining moving-average filtering, thresholding, and collar correction. **Results:** On the public CHB-MIT dataset and the clinical SH-SDU dataset, the proposed method achieved average segment-level sensitivities of 98.68% and 92.05%, specificities of 98.33% and 97.53%, accuracies of 98.49% and 96.37%, and AUC values of 97.26% and 92.89%, respectively. In event-level evaluation, the average sensitivities were 99.13% and 96.08%, with false detection rates of 0.88/h and 0.69/h, respectively. Further multi-stage ablation experiments together with t-SNE and Grad-CAM visualizations provided qualitative and experimental support for the design rationale of the joint power-phase input and the hybrid 3D-CNN-ViT architecture. **Conclusions:** The proposed framework effectively exploits the complementary discriminative value of power and phase information in epileptic EEG and demonstrates strong detection performance under patient-specific evaluation on both public and clinically collected datasets.

## 1. Introduction

Epilepsy is a chronic neurological disorder characterized by recurrent seizures that arise from abnormal excessive or synchronous neuronal activity in the brain, with marked clinical heterogeneity and long-term disease burden [1]. The World Health Organization reports that approximately 50 million people worldwide are affected by epilepsy, and epidemiological studies further indicate that the lifetime prevalence of epilepsy is about 7.60 per 1000 [2,3]. Beyond the recurrent seizures themselves, epilepsy is frequently accompanied by depression, anxiety, and other neuropsychiatric comorbidities, which often interact with seizure control and further reduce patients’ quality of life [4]. In patients with drug-resistant epilepsy, the risk of sudden unexpected death in epilepsy is significantly increased and is believed to be closely associated with postictal cardiopulmonary dysfunction, autonomic imbalance, and impaired brainstem regulation [5].

Electroencephalography (EEG), owing to its ability to record changes in brain activity with high temporal resolution, remains one of the most important auxiliary tools in epilepsy diagnosis [6]. Recent clinical studies have further highlighted the diagnostic value of EEG in neurological workups, particularly for confirming or excluding epileptic seizures in patients with unexplained neurological symptoms [7]. Under long-term monitoring conditions, EEG provides critical evidence for seizure-event capture and subsequent therapeutic evaluation [8]. However, conventional EEG interpretation depends heavily on clinical expertise, and disagreement among readers may still arise in the identification of abnormal patterns and the delineation of event boundaries [9,10]. Therefore, there is a strong need to develop robust, automated seizure detection methods with clinical deployment potential.

Early studies on automatic seizure detection from EEG mainly relied on the combination of handcrafted features and conventional machine-learning models. These approaches typically focused on morphological, frequency-domain, and nonlinear dynamical features, which were then used together with classifiers such as Bayesian linear discriminant analysis (BLDA) and support vector machines (SVMs) for detection [11]. With the rapid development of deep learning, researchers began to employ CNNs, RNNs, LSTMs, and their variants to automatically extract discriminative representations from raw EEG signals or transformed features, thereby reducing the prior constraints imposed by manual feature engineering [12,13]. Recent reviews have also summarized the taxonomy, opportunities, and remaining challenges of machine-learning-based epileptic seizure detection, highlighting the importance of robust feature extraction, reliable classification, and clinically applicable evaluation protocols [14]. Recent EEG-based seizure detection studies have also emphasized that artifact removal and effective preprocessing remain important for improving diagnostic accuracy in epilepsy-related EEG analysis [15]. Nevertheless, inter-patient variability, artifact contamination, and the lack of standardized evaluation protocols still limit the stable deployment of these methods in real clinical settings [16,17].

For epileptic EEG, a typical nonstationary signal, time–frequency analysis provides an important means of characterizing seizure-related dynamics. In recent years, the short-time Fourier transform (STFT), the S-transform, and their variants have been widely used in seizure detection and prediction studies and combined with deep models in various ways [18,19]. However, different time–frequency transforms differ in their ability to represent temporal resolution, frequency resolution, and transient structures, which in turn affects how well subsequent models can exploit epileptic features. By contrast, the continuous wavelet transform (CWT), through its multiscale analysis mechanism, preserves higher temporal resolution in the high-frequency range and better frequency resolution in the low-frequency range, making it more suitable for describing the complex coexistence of fast transients and slow rhythms in epileptic EEG [20,21]. At the same time, studies combining wavelet-based time–frequency representations with deep learning have continued to increase, further demonstrating the strong compatibility between wavelet-domain features and deep representation learning [22].

In addition to power features, the potential value of phase-related information in epileptic EEG analysis has also attracted increasing attention. Previous studies have shown that phase-amplitude coupling (PAC) and other phase-related indices are closely associated with seizure evolution and abnormal brain-network activity [23], suggesting that phase information can provide complementary discriminative cues for EEG-based seizure detection. Meanwhile, the strong capability of Transformers to model long-range dependencies in natural language processing (NLP) has driven their extension to vision tasks, and the vision transformer (ViT) has consequently become an important representative architecture for image analysis [24,25]. In EEG analysis, an increasing number of studies have begun to explore Transformers and hybrid Transformer-based models in motor imagery, emotion recognition, and seizure detection, indicating clear complementarity between convolutional modeling and self-attention modeling [26,27,28].

Despite continuous advances in this field, two issues still merit further investigation. First, although recent studies have incorporated phase and power information into EEG-based seizure detection, most existing approaches use separate two-dimensional streams or relatively simple fusion strategies. Such designs may not fully capture the joint interactions among frequency, time, channel, and modality dimensions in multichannel EEG time–frequency representations. Second, existing networks are often biased toward either local convolutional modeling or isolated global attention modeling, and their ability to jointly mine local time–frequency–channel patterns and higher-level global dependencies remains limited. To address these limitations, we propose a CWT-based 3D-CNN-ViT hybrid framework for seizure detection. Specifically, power spectrograms and phase spectrograms are constructed from multichannel EEG segments and fused along the depth dimension into a unified power-phase volume. A 3D-CNN is then used to extract local frequency–time–channel–modality coupling features, and a ViT is further employed to model higher-level global dependencies, thereby enabling effective identification of seizure segments. The proposed method is evaluated on the public CHB-MIT dataset and a clinically collected SH-SDU dataset, and its design rationale and interpretability are further discussed through multi-stage ablation experiments and visualization analyses.

The main contributions of this study can be summarized as follows:

- A joint modeling strategy based on CWT-based power spectrograms and phase spectrograms is proposed, and the two representations are fused into a unified power-phase volume to preserve complementary epileptic discriminative information more effectively.
- A hybrid 3D-CNN-ViT architecture for seizure detection is designed, in which the 3D-CNN extracts local time–frequency–channel patterns and the ViT models global dependencies across time and feature dimensions, thereby enhancing the representation of complex nonstationary EEG.
- The proposed method is systematically evaluated on the public CHB-MIT dataset and the clinically collected SH-SDU dataset, and its detection performance is assessed at both the segment level and the event level under a patient-specific setting.

To improve readability, an abbreviation index is provided in Abbreviations Section.

## 2. EEG Datasets

### 2.1. CHB-MIT Database

The publicly available CHB-MIT scalp EEG database was used as the primary benchmark dataset in this study. Collected at Boston Children’s Hospital and released through PhysioNet by the Massachusetts Institute of Technology, this dataset is one of the most widely used public long-term scalp EEG resources for seizure detection research [29,30]. It contains long-term scalp EEG recordings from 24 patients with epilepsy. All signals were sampled at 256 Hz with 16-bit resolution. The recordings typically include 18-23 bipolar channels, and electrode placement follows the international 10-20 system. The seizure labels provided with the CHB-MIT database were used as the ground truth for model training and evaluation [30,31].

Because channel configurations are not identical across cases, only the 18 bipolar channels shared by all patients were retained to ensure a uniform input dimensionality across subjects: Fp1-F7, F7-T7, T7-P7, P7-O1, Fp1-F3, F3-C3, C3-P3, P3-O1, Fp2-F4, F4-C4, C4-P4, P4-O2, Fp2-F8, F8-T8, T8-P8, P8-O2, Fz-Cz, and Cz-Pz. This step ensured dimensional consistency of the input samples across patients and provided a consistent input format for patient-specific model training and evaluation [31].

Table 1 summarizes the statistics of the CHB-MIT cases used in this study. Patient chb16 was excluded from the experimental pipeline because most of its seizure events were extremely short, making reliable patient-specific training and event-level evaluation difficult. Therefore, 23 CHB-MIT cases were included for model training, testing, and final performance evaluation. Overall, the included cases comprise 960.93 h of EEG recordings and 174 seizure events, of which 32 seizure events were used for training. The total durations of seizure and non-seizure EEG used for model training were 29.25 min and 146.27 min, respectively. For each patient, a patient-specific data partition strategy was adopted. Earlier annotated seizure events were used for model training, whereas the remaining later seizure events were reserved for testing. The split was performed at the seizure-event level rather than by randomly dividing overlapped EEG segments. To mitigate the class imbalance between seizure and non-seizure samples in the training set, overlap-based upsampling was applied only to the selected training seizure events, thereby increasing the number of ictal training segments. Note that the training and testing sets were strictly separated, and no EEG segment derived from any test seizure event was used during model training.

### 2.2. SH-SDU Database

To further validate the proposed method, we additionally used a clinical scalp EEG database collected at the Second Hospital of Shandong University (SH-SDU). The database consists of continuous video-EEG monitoring recordings from adult patients with epilepsy. The raw signals were acquired at 500 Hz using an 18-channel monopolar montage. Electrode placement followed the international 10–20 system, with the reference electrode located at the midpoint of the line connecting Fz and Cz. The recorded channels included Fp1, Fp2, F3, F4, C3, C4, P3, P4, O1, O2, F7, F8, T3, T4, T5, T6, A1, and A2. For the SH-SDU dataset, the seizure events marked in this dataset were determined by two trained clinical experts according to EEG evolution and the corresponding clinical manifestations [32]. These expert-defined seizure labels were used as the ground truth for the proposed automatic detection framework.

Table 2 summarizes the statistics of the SH-SDU dataset used in this study. A total of 6 cases were ultimately included, comprising 88.18 h of continuous EEG recordings and 76 seizure events, of which 12 were used for training. Patient ages ranged from 28 to 76 years, and the mean seizure duration ranged from 34.67 to 240.60 s, indicating substantial inter-subject variability. The total durations of seizure and non-seizure EEG used for model training were 14.30 min and 71.50 min, respectively. The SH-SDU database followed the same patient-specific and event-level partition strategy as CHB-MIT database. For each patient, earlier annotated seizure events were used for training, whereas the remaining later seizure events were reserved for testing. To mitigate class imbalance in the training set, overlap-based upsampling was applied only to the selected training seizure events. Note that the training and testing event sets were strictly separated, and no EEG segment derived from any test seizure event was used during model training.

## 3. Methods

**Figure 1:**
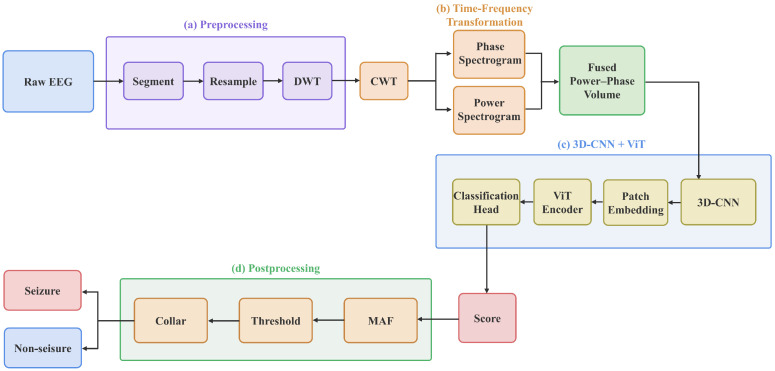
Workflow of the proposed seizure detection framework. The preprocessing module includes signal segmentation, EEG resampling, and the discrete wavelet transform (DWT). The time–frequency module uses the continuous wavelet transform (CWT) to generate phase and power spectrograms and to construct fused features. The deep learning module integrates a 3D-CNN, patch embedding, a ViT encoder, and a classification head. The postprocessing module consists of moving-average filtering (MAF), thresholding, and collar correction.

As shown in Figure 1, the proposed seizure detection framework consists of four major components: preprocessing, time–frequency transformation, deep learning-based modeling, and postprocessing. The preprocessing stage includes EEG segmentation, resampling, and the discrete wavelet transform (DWT). In the time–frequency stage, the continuous wavelet transform (CWT) is used to generate power and phase spectrograms and to construct a fused power-phase representation. The deep learning stage is composed of a 3D-CNN, a patch embedding module, a ViT encoder, and a classification head for distinguishing seizure from non-seizure states. The postprocessing stage then applies moving-average filtering (MAF), thresholding, and collar correction to the model output scores to obtain the final detection results.

### 3.1. Preprocessing

Before seizure detection and model training, the raw multichannel EEG recordings were subjected to a unified preprocessing pipeline to reduce the influence of electrooculographic (EOG) artifacts, electromyographic (EMG) interference, and other noise on subsequent time–frequency representation and classification [14]. Specifically, all EEG recordings were unified to a sampling rate of 256 Hz when necessary. In particular, the original 500-Hz recordings in the SH-SDU dataset were resampled to 256 Hz before segmentation using the MATLAB R2024b resample routine, which applies an anti-aliasing low-pass FIR filter before resampling [33]. The EEG signals were then segmented into 4-s analysis windows, and each segment contained 1024 samples.

Each 4-s EEG segment was then processed using the discrete wavelet transform (DWT) with a Daubechies-4 (db4) wavelet. DWT has been widely used for multiscale decomposition and denoising of EEG signals because it preserves informative neural activity while enhancing the representation of features in different frequency bands [34]. Owing to its strong ability to characterize transient and irregular EEG fluctuations, the db4 wavelet is commonly employed for EEG analysis and extraction of epilepsy-related patterns [35]. After unifying the sampling rate to 256 Hz, the corresponding Nyquist frequency was 128 Hz. Previous studies have shown that seizure-related scalp EEG activities are commonly analyzed within conventional low-frequency ranges, and wavelet-based time–frequency representations have been widely used for epileptic EEG analysis [36,37]. The signal was decomposed into five sets of detail coefficients and one set of approximation coefficients, corresponding to the 64–128 Hz (D1), 32–64 Hz (D2), 16–32 Hz (D3), 8–16 Hz (D4), 4–8 Hz (D5), and 0–4 Hz (A5) bands, respectively. Therefore, the D3, D4, and D5 components were retained to reconstruct the 4–32 Hz band-limited signal [32,38]. This reconstructed band covers the theta, alpha, and beta rhythms commonly used in scalp EEG seizure-detection studies while reducing very-low-frequency drift and high-frequency noise components. The reconstructed 4–32 Hz band, together with the 1–40 Hz CWT frequency range used in the proposed framework, remains well below the 128 Hz Nyquist frequency. The DWT-preprocessed EEG segments were then fed into the CWT module to generate power and phase spectrograms for subsequent deep learning-based training and detection.

### 3.2. CWT-Based Power-Phase Time–Frequency Representation

The continuous wavelet transform (CWT) can jointly characterize the temporal evolution and frequency distribution of a signal, making it particularly suitable for nonstationary signals such as epileptic EEG [39]. For a time-domain signal x(t), the CWT can be expressed as:

(1) $$W_{x}\left(a,b\right)=\frac{1}{\sqrt{a}}\int_{-\infty }^{+\infty }x\left(t\right)\psi^{*}\left(\frac{t-b}{a}\right)\;dt.$$

where *a* is the scale parameter that controls the dilation and contraction of the wavelet function. Larger values of *a* correspond to a wider time window and are more suitable for characterizing low-frequency components, whereas smaller values of *a* correspond to a narrower time window and are better suited for capturing high-frequency transient details. The parameter *b* denotes translation and determines the position of the wavelet along the time axis, while $\psi^{*}\left(\cdot \right)$ denotes the complex conjugate of the mother wavelet.

In this study, the complex Morlet wavelet was adopted as the mother wavelet for CWT, and its expression is given by:

(2) $$\psi \left(t\right)=\frac{1}{\sqrt{\pi f_{b}}}\exp\left(2\pi if_{c}t\right)\exp\left(-\frac{t^{2}}{f_{b}}\right).$$

where $f_{b}$ is the bandwidth parameter and $f_{c}$ is the center-frequency parameter. The complex Morlet wavelet provides favorable time–frequency localization while preserving amplitude information and providing phase information; it is therefore well suited to EEG time–frequency analysis [40]. In our experiments, $f_{b}=1$ and $f_{c}=1$ were used, corresponding to the cmor1-1 wavelet, to balance time and frequency resolution. Accordingly, the complex coefficients obtained after CWT can be written as:

(3) $$W_{x}\left(a,b\right)=A\left(a,b\right)e^{i\phi (a,b)}.$$

where A(a,b) denotes amplitude information and ϕ(a,b) denotes phase information. Based on the complex coefficients $W_{x}\left(a,b\right)$, the power spectrogram and phase spectrogram are defined as follows:

(4) $$|W_{x}{\left(a,b\right)|}^{2}=W_{x}\left(a,b\right)\cdot W_{x}^{*}\left(a,b\right).$$

(5) ϕ(a,b)=atan2Im(Wx(a,b)),Re(Wx(a,b)).

**Figure 2:**
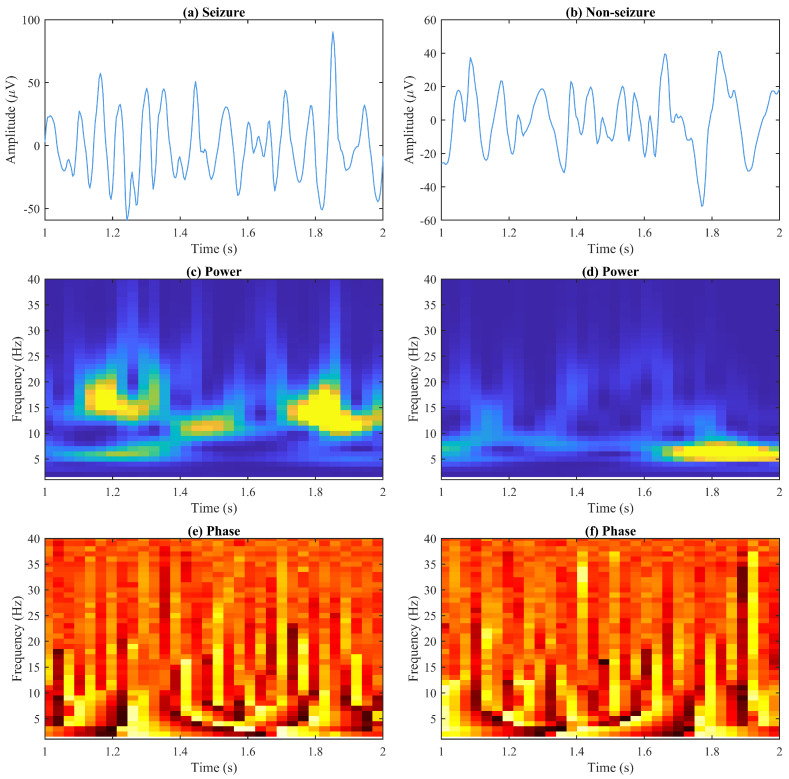
Time-domain waveforms and CWT time–frequency representations of seizure and non-seizure segments. (**a**,**b**) Time-domain EEG waveforms of seizure and non-seizure segments over the 1–2 s interval. (**c**,**d**) Corresponding power spectrograms. (**e**,**f**) Corresponding phase spectrograms. Both the power and phase spectrograms were obtained using the continuous wavelet transform (CWT), with a frequency range of 1–40 Hz along the vertical axis.

CWT was applied to each 4-s EEG segment using a frequency range of 1–40 Hz and a frequency resolution of 1 Hz. Because each segment contains 1024 temporal samples, the time axis was averaged every 8 points to reduce computational cost while preserving the dominant time–frequency structure, thereby compressing the temporal dimension from 1024 to 128. For each EEG channel, a corresponding power spectrogram and phase spectrogram were obtained; for an 18-channel EEG segment, this yielded a total of 36 time–frequency maps, which served as the input basis for the subsequent deep learning model. Recent studies have likewise shown that CWT-based time–frequency representations are highly compatible with deep learning models and can effectively support automatic identification of epilepsy-related patterns [41]. As illustrated in Figure 2, seizure and non-seizure segments exhibit clear differences not only in the time-domain waveforms but also in their corresponding CWT time–frequency representations, and the power and phase spectrograms characterize seizure-related features from complementary perspectives.

### 3.3. Hybrid 3D-CNN–ViT Architecture for Seizure Detection

**Figure 3:**
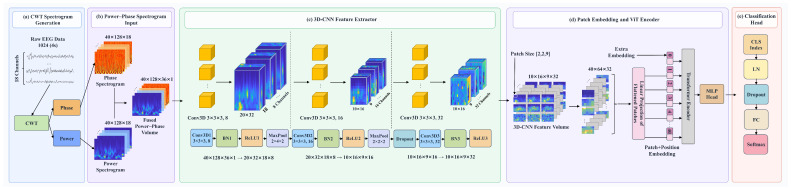
Schematic of the hybrid 3D-CNN-ViT architecture. (**a**) CWT power and phase spectrograms generated from raw 4-s, 18-channel EEG segments. (**b**) Power and phase spectrograms are represented as time–frequency volumes of size 40 × 128 × 18 and then concatenated along the depth dimension to form a fused power-phase volume of size 40 × 128 × 36 × 1. (**c**) The 3D-CNN feature extractor consists of three convolutional blocks. The first two are followed by batch normalization, ReLU activation, and 3D max pooling; dropout is inserted after the second block; the third block contains convolution, batch normalization, and ReLU activation only. (**d**) The convolutional feature volume is mapped into a token sequence by patch embedding and then passed, together with a learnable CLS token and positional encoding, into a single-layer ViT encoder. (**e**) The classification head outputs seizure and non-seizure probabilities through CLS indexing, layer normalization, dropout, a fully connected layer, and softmax.

We propose a hybrid 3D-CNN-ViT seizure detection architecture based on a joint power-phase time–frequency representation, which combines CWT-based EEG features with convolutional neural networks and a vision transformer. For each 4-s EEG segment with 18 channels, power spectrograms and phase spectrograms of size 40 × 128 × 18 are first generated, as illustrated in Figure 3a. The two representations are then concatenated along the depth dimension to form a fused power-phase volume of size 40 × 128 × 36 × 1 that serves as the network input, as shown in Figure 3b. This fused representation is first processed by a 3D-CNN feature extractor to progressively learn local time–frequency–channel patterns through three 3D convolutional layers. The resulting convolutional feature volume is then mapped into a token sequence via patch embedding and fed into a single-layer ViT encoder to model higher-level global dependencies. Finally, the classification head outputs seizure and non-seizure predictions based on the learned discriminative features.

#### 3.3.1. 3D-CNN Feature Extractor

As shown in Figure 3c, the 3D-CNN feature extractor takes the fused power-phase volume of size 40 × 128 × 36 × 1 as input, where 40, 128, and 36 correspond to the frequency, time, and modality-channel depth dimensions, respectively, and the last dimension denotes a single input channel. Unlike conventional 2D convolution, which extracts local patterns only within a plane, 3D convolution jointly models the frequency, time, and modality-channel depth dimensions and is therefore more suitable for learning local time–frequency–channel coupling patterns from EEG time–frequency representations [42]. Let $X\in R^{H\times W\times D\times C}$ denote the input feature volume. The 3D convolution operation can be written as:

(6) $$Y_{k}\left(i,j,l\right)=\sum_{c=1}^{C}\sum_{u=-r_{h}}^{r_{h}}\sum_{v=-r_{w}}^{r_{w}}\sum_{m=-r_{d}}^{r_{d}}W_{k,c}\left(u,v,m\right)\;X\left(i+u,j+v,l+m,c\right)+b_{k}.$$

where $W_{k,c}$ and $b_{k}$ denote the weights and bias of the *k*-th convolution kernel for the *c*-th input channel, respectively. The parameters $r_{h}$, $r_{w}$, and $r_{d}$ denote the half kernel sizes along the three spatial dimensions. For clarity, padding and stride operations are omitted in this formulation.

The first convolutional block receives an input feature volume of size 40 × 128 × 36 × 1 and uses eight 3 × 3 × 3 kernels to extract shallow local features, producing an output of size 40 × 128 × 36 × 8. The output features are then passed through batch normalization and ReLU activation to stabilize the feature distribution and enhance the nonlinear expressive capacity of the network. The ReLU activation function is defined as:

(7) ReLU(x)=max(0,x).

A 3D max-pooling layer with a stride of [2,4,2] is then used to downsample the feature volume, reducing the output size to 20 × 32 × 18 × 8. The second convolutional block also uses 3 × 3 × 3 kernels and increases the number of feature channels from 8 to 16. After convolution, batch normalization, and ReLU activation, the feature size remains 20 × 32 × 18 × 16; a subsequent 3D max-pooling layer with stride [2,2,2] further compresses the features, yielding an output of size 10 × 16 × 9 × 16. After the second pooling layer, a dropout layer with a dropout rate of 0.2 is introduced to mitigate overfitting during network training [43]. Given an input feature vector *z*, dropout can be expressed as:

(8) $$\tilde{z}=\frac{M⊙z}{1-p},\;M∼\mathrm{Bernoulli}\left(1-p\right).$$

where *p* denotes the dropout rate, *M* is a Bernoulli mask with keep probability 1−p, and ⊙ denotes element-wise multiplication. This formulation corresponds to inverted dropout, in which the retained activations are scaled by 1/(1−p) during training; during prediction, the dropout layer acts as an identity mapping.

After dropout, the third convolutional block continues to use 3 × 3 × 3 kernels and further expands the number of output channels to 32. This block is still followed by batch normalization and ReLU activation, but no additional pooling is applied. As a result, the output size remains 10 × 16 × 9 × 32, providing the input for the subsequent patch embedding and ViT encoder.

The detailed layer-wise configuration of the proposed 3D-CNN feature extractor is provided in Table 3.

#### 3.3.2. ViT Encoder for Classification

As shown in Figure 3d, the convolutional feature volume obtained after the 3D-CNN module has a size of 10 × 16 × 9 × 32. To map this 3D feature representation into a sequence suitable for Transformer-based modeling, patch embedding is used to partition the feature volume and project each patch linearly. Specifically, the patch size is set to [2,2,9], so the input feature volume is divided into 5 × 8 × 1 = 40 nonoverlapping patches. Each patch covers a local 3D region of size 2 × 2 × 9 and includes all 32 feature channels, resulting in 2×2×9×32=1152 values after flattening. A linear projection layer then maps each 1152-dimensional patch vector to a 64-dimensional token, thereby forming a token sequence of length 40.

To enhance global discriminative modeling, a learnable classification token (CLS token) is appended to the beginning of the token sequence, and a learnable positional encoding is added to preserve the spatial positions of patches within the original convolutional feature volume. Let the token sequence after patch embedding be $Z_{p}\in R^{40\times 64}$, the CLS token be $z_{\mathrm{cls}}\in R^{1\times 64}$, and the positional encoding be $E_{\mathrm{pos}}\in R^{41\times 64}$. The input sequence to the ViT encoder can then be written as:

(9) $$X_{0}=\left[z_{\mathrm{cls}};Z_{p}\right]+E_{\mathrm{pos}}.$$

where [·;·] denotes concatenation along the sequence dimension. After this operation, the input to the ViT encoder has a size of 41 × 64.

**Figure 4:**
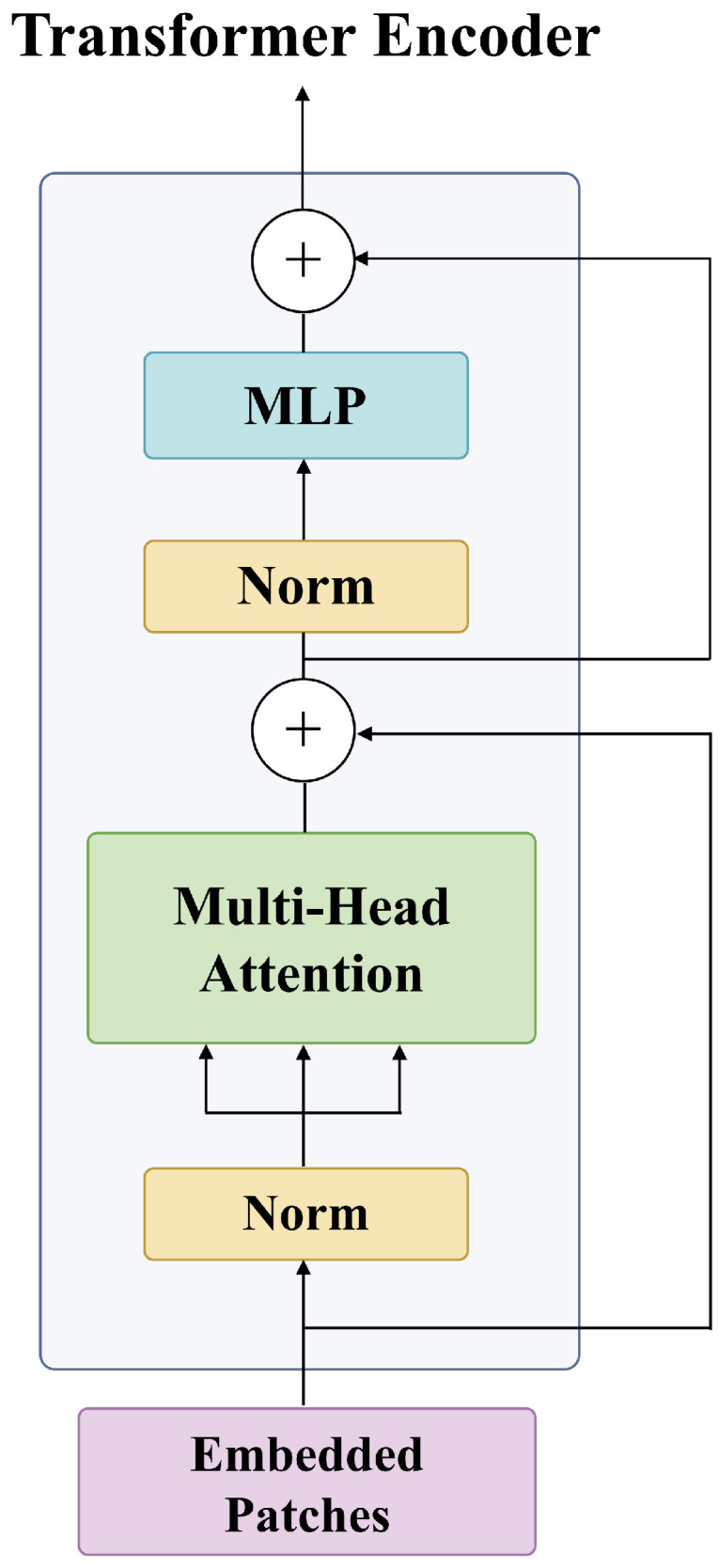
Schematic of the Transformer encoder. The encoder consists of a layer normalization module, a multi-head self-attention module, and a multilayer perceptron module.

As shown in Figure 4, a single standard Transformer encoder is used to model global dependencies in the token sequence [24,25]. The encoder consists of two parts: (1) layer normalization, multi-head self-attention, and residual connection, and (2) layer normalization, a multilayer perceptron (MLP), and residual connection. Let the input sequence be $X_{0}\in R^{N\times D}$, where N=41 is the sequence length and D=64 is the embedding dimension. Multi-head self-attention first maps the input sequence to Query, Key, and Value:

(10) $$Q=X_{0}W_{Q},\;K=X_{0}W_{K},\;V=X_{0}W_{V}.$$

For the *i*-th attention head, the output can be written as:

(11) $${\mathrm{head}}_{i}=\mathrm{softmax}\left(\frac{Q_{i}K_{i}^{T}}{\sqrt{d_{h}}}\right)V_{i}.$$

where $d_{h}=D/h$ denotes the feature dimension of each head and *h* is the number of attention heads. In this study, h=4, so each head has dimension $d_{h}=16$. The outputs of all attention heads are concatenated along the feature dimension and then linearly projected to obtain the final multi-head self-attention output:

(12) $$\mathrm{MSA}\left(X_{0}\right)=\mathrm{Concat}\left({\mathrm{head}}_{1},\ldots ,{\mathrm{head}}_{h}\right)W_{O}.$$

To stabilize training, a pre-norm residual structure is adopted. After layer normalization and multi-head self-attention, the intermediate output can be expressed as:

(13) $$Z_{1}=X_{0}+\mathrm{MSA}\left(\mathrm{LayerNorm}\left(X_{0}\right)\right).$$

The encoder then applies a multilayer perceptron (MLP) for further nonlinear feature transformation. The MLP used in this study consists of two fully connected layers with an input dimension of 64, a hidden dimension of 128, and an output dimension restored to 64. A GELU activation function and dropout with a rate of 0.1 are inserted between the two layers to enhance nonlinear representation and suppress overfitting. Its formulation is:

(14) $$\mathrm{MLP}\left(Z_{1}\right)=\mathrm{Dropout}\left({\mathrm{FC}}_{2}\left(\mathrm{Dropout}\left(\mathrm{GELU}({\mathrm{FC}}_{1}\left(Z_{1}\right))\right)\right)\right).$$

The final encoder output is given by:

(15) $$X_{\mathrm{out}}=Z_{1}+\mathrm{MLP}\left(\mathrm{LayerNorm}\left(Z_{1}\right)\right).$$

As shown in Figure 3e, in the classification stage only the CLS token at the beginning of the sequence is retained as the final discriminative representation. It is extracted from the full sequence by a one-dimensional indexing operation. Let the output CLS token of the encoder be $z_{\mathrm{cls}}^{*}\in R^{1\times 64}$. The classification head first applies layer normalization, followed by dropout with a rate of 0.3 to reduce overfitting; the result is then passed to a fully connected layer with output dimension 2 for category mapping, and finally softmax is used to obtain the predicted probabilities of seizure and non-seizure:

(16) $$\hat{y}=\mathrm{softmax}\left(\mathrm{FC}(\mathrm{Dropout}\left(\mathrm{LayerNorm}\left(z_{\mathrm{cls}}^{*}\right)\right))\right).$$

### 3.4. Model Training

A patient-specific training and testing strategy was adopted, whereby training and test sets were constructed separately for each subject. The model was trained end-to-end in a supervised manner. Network parameters were iteratively updated using the Adam optimizer [44], with cross-entropy loss as the optimization objective [45]. Let $y_{i}$ denote the ground-truth label vector of the *i*-th sample and ${\hat{y}}_{i}$ denote the predicted probability vector produced by the model. The cross-entropy loss is defined as:

(17) $$L_{\mathrm{CE}}=-\frac{1}{N}\sum_{i=1}^{N}\sum_{c=1}^{2}y_{i,c}\log\left({\hat{y}}_{i,c}\right).$$

where *N* denotes the number of samples in a mini-batch and *c* denotes the class index. Model parameters are optimized end-to-end by minimizing this loss function.

In the specific training configuration, the initial learning rate was set to $2\times 10^{-4}$ and the maximum number of training epochs was set to 100. To balance rapid convergence in the early stage with stable fine-tuning in the later stage, a piecewise learning-rate decay schedule was adopted, in which the learning rate was reduced by a fixed decay factor every 20 epochs until it reached $2\times 10^{-5}$ at the end of training. In addition, L2 regularization with a coefficient of $1\times 10^{-3}$ was introduced to alleviate overfitting, and the mini-batch size was set to 128 to balance stable gradient estimation, GPU memory consumption, and training efficiency.

**Figure 5:**
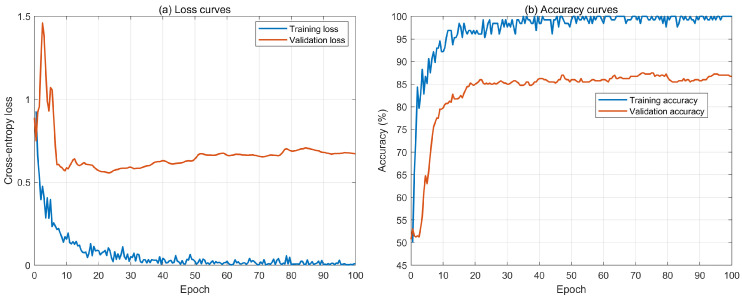
Training and validation loss/accuracy curves for CHB-MIT Patient 12. The held-out validation subset consisted of 200 seizure segments and 200 non-seizure segments that were not used for model parameter updating. The validation accuracy remained stable at approximately 86–87% in the later epochs.

To further examine the training behavior of the proposed model, an additional held-out validation analysis was performed on CHB-MIT Patient 12. Specifically, 200 seizure segments and 200 non-seizure segments that were not used for model parameter updating were selected to construct a balanced validation subset. As shown in Figure 5, the validation accuracy increased rapidly in the early training stage and remained stable at approximately 86–87% in the later epochs. Although the validation loss showed mild late-stage fluctuations, no continuous decrease in validation accuracy was observed.

### 3.5. Postprocessing

**Figure 6:**
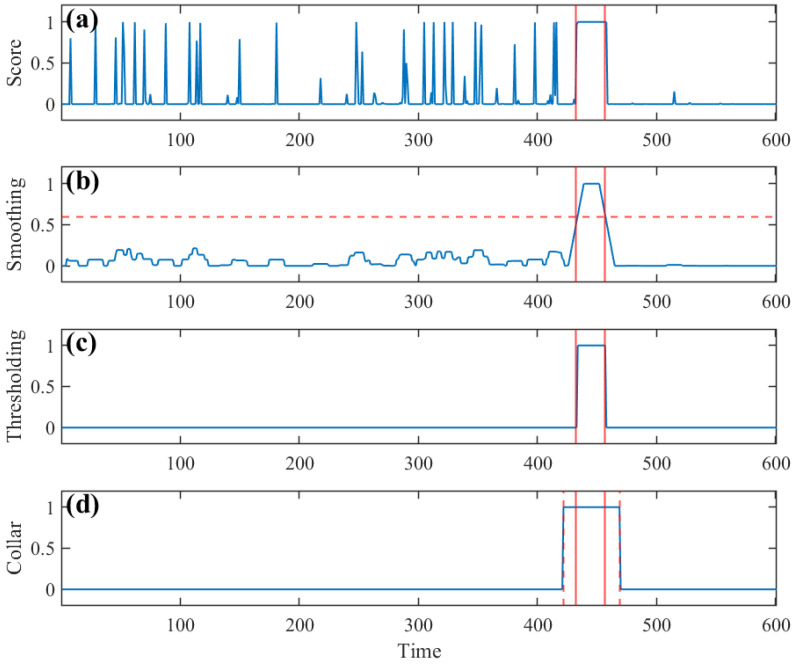
Illustration of the postprocessing procedure applied to the model output scores. (**a**) Raw output score sequence of the model. (**b**) Score sequence after smoothing with moving-average filtering (MAF), where the red horizontal dashed line indicates the threshold. (**c**) Binary decisions after thresholding. (**d**) Final detection results after additional collar correction. In the figure, the red vertical solid lines indicate expert-annotated seizure intervals, and the red vertical dashed lines indicate the collar-expanded detection boundaries.

To further improve temporal continuity and discriminative reliability, a postprocessing procedure was designed based on the raw output scores of the model, as illustrated in Figure 6. This procedure includes three steps: score smoothing, threshold-based binarization, and collar correction. Similar postprocessing strategies have been used in epilepsy-related automatic detection studies to reduce false alarms caused by isolated high-score segments and to improve temporal consistency of event boundaries [32,46].

Let s(t) denote the raw output score of the model at time *t*. A symmetric moving-average filter is first applied to the score sequence to suppress high-frequency fluctuations and enhance temporal continuity of seizure segments. The smoothed score $\bar{s}\left(t\right)$ can be written as:

(18) $$\bar{s}\left(t\right)=\frac{1}{2N+1}\sum_{k=-N}^{N}s\left(t+k\right)$$

where 2N+1 denotes the window length of the moving-average filter and *N* is the half-window size. This operation effectively suppresses isolated spikes and short-term noise perturbations in the model output, thereby reducing the influence of random fluctuations on the detection results.

After smoothing, the continuous score sequence is compared with a threshold Thr to obtain binary detection results. Specifically, when $\bar{s}\left(t\right)\geq \mathrm{Thr}$, the corresponding segment is classified as seizure; otherwise, it is classified as non-seizure. The decision rule can be written as:

(19) $$\hat{y}\left(t\right)=\left\{\begin{array}{cc} 1, & \bar{s}\left(t\right)\geq \mathrm{Thr},\\ 0, & \bar{s}\left(t\right)<\mathrm{Thr}. \end{array}\right.$$

where $\hat{y}\left(t\right)$ denotes the thresholded binary label, and 1 and 0 correspond to seizure and non-seizure, respectively. The threshold Thr is not a fixed constant. Instead, it is optimized on a patient-specific basis to achieve a more appropriate balance between sensitivity and specificity.

To compensate for potential boundary shrinkage or detection delay introduced by smoothing, collar correction is further applied to the thresholded results. If the onset and offset positions of a detected seizure segment in the binary sequence are denoted by $t_{s}$ and $t_{e}$, respectively, the corrected detection interval is expanded to:

(20) $$[t_{s}-K,\;t_{e}+K].$$

where *K* denotes the number of points by which the interval is extended before seizure onset and after seizure termination. This strategy allows transition regions to be covered more fully without substantially increasing false detections, thereby improving temporal alignment between predicted boundaries and expert annotations. In this study, the moving-average window parameter *N*, the threshold Thr, and the collar expansion parameter *K* were all optimized in a patient-specific manner to obtain the best detection performance.

### 3.6. Performance Metrics and Evaluation Setup

All experiments were conducted in MATLAB R2024b on a workstation equipped with an Intel Core i9-14900K processor and an NVIDIA GeForce RTX 3090 GPU. Data preprocessing and time–frequency feature construction were mainly carried out on the CPU, whereas training and inference of the proposed 3D-CNN-ViT network were performed on the GPU. To comprehensively evaluate model performance, analyses were conducted at both the segment level and the event level. Segment-level evaluation used sensitivity, specificity, and accuracy, defined as follows:

(21) $$\mathrm{Sensitivity}=\frac{TP}{TP+FN}\times 100\%.$$

(22) $$\mathrm{Specificity}=\frac{TN}{TN+FP}\times 100\%.$$

(23) $$\mathrm{Accuracy}=\frac{TP+TN}{TP+TN+FP+FN}\times 100\%.$$

where TP, TN, FP, and FN denote correctly identified seizure segments, correctly identified non-seizure segments, non-seizure segments incorrectly classified as seizures, and missed seizure segments, respectively. Event-level evaluation employed event-based sensitivity and the false detection rate (FDR). Event-based sensitivity denotes the proportion of expert-annotated seizure events that were successfully detected, whereas FDR denotes the number of false-positive seizure events per unit time. These two metrics have been widely used in event-level evaluation of seizure detection tasks [47]. They are defined as follows:

(24) $$\mathrm{Event}\text{-}\mathrm{based}\;\mathrm{Sensitivity}=\frac{N_{d}}{N_{e}}\times 100\%.$$

(25) $$\mathrm{FDR}=\frac{N_{\mathrm{fp}}}{T_{\mathrm{test}}}.$$

where $N_{e}$ and $N_{d}$ denote the numbers of expert-annotated seizure events and detected seizure events, respectively, $N_{\mathrm{fp}}$ denotes the number of false-positive events, and $T_{\mathrm{test}}$ denotes the total duration of the test recording in hours. For segment-level and event-level operating-point metrics, the post-processing parameters, including the smoothing window length *N*, the decision threshold Thr, and the collar expansion length *K*, were treated as patient-specific operating settings to control the sensitivity–false-alarm trade-off. These parameters were selected using non-test data only. Specifically, for each patient, the training portion and an additional 20-min seizure-free EEG calibration segment were used for post-processing parameter calibration. The calibration segment was selected from non-ictal EEG periods and was used only to adjust the post-processing parameters according to the patient’s baseline non-ictal score distribution. It was not used for network training, did not update any model weights, did not overlap with any seizure event or any EEG segment used for training/testing, and was excluded from sample generation and upsampling procedures. After calibration, *N*, Thr, and *K* were fixed before final test evaluation, and the test set was reserved solely for final performance reporting. To further assess model performance under a unified post-processing setting, the receiver operating characteristic (ROC) curve and the area under the curve (AUC) were additionally calculated using fixed smoothing and collar windows for all patients. Specifically, when calculating AUC, the smoothing window length and collar length were both fixed at 24 s. In addition, because AUC is a threshold-independent metric, it reflects classifier performance across all possible decision thresholds rather than relying on a single patient-specific Thr [48]. The closer the AUC is to 1, the stronger the discriminative performance of the model; an AUC close to 0.5 indicates almost no effective discrimination. All segment-level, event-level, and AUC results were finally summarized across the two datasets.

## 4. Results

### 4.1. Results on CHB-MIT Database

The detection results on the CHB-MIT dataset are presented in Table 4. Across the 23 patients included in the final evaluation, the proposed method demonstrated stable segment-level performance, with average sensitivity, specificity, and accuracy of 98.68 ± 2.97%, 98.33 ± 2.31%, and 98.49 ± 2.10%, respectively. Among these patients, segment-level sensitivity reached 100% in 15 cases, and accuracy exceeded 99% in 16 cases, indicating that the model effectively distinguished seizure from non-seizure segments in most patients.

At the event level, the method also showed potential practical utility. As summarized in Table 4, after excluding seizures used for training, the 23 patients included in the final statistics contained 142 expert-annotated seizure events, of which 141 were successfully detected. The macro-averaged event-based sensitivity across patients reached 99.13 ± 4.17%, while the pooled event-level detection ratio was 141/142 = 99.30%. Among the 23 patients, 22 achieved 100% event-based sensitivity, and only 1 patient did not have all seizure events detected. Meanwhile, the average false detection rate (FDR) was 0.88 ± 1.37/h, and 16 patients had an FDR below 0.5/h. These results indicate that the proposed method can maintain a high seizure detection rate while effectively controlling false-positive events.

To further assess the overall discriminative ability of the model output scores for seizure and non-seizure states, the AUC was also computed for each patient. As shown in Table 4, the average AUC reached 97.26 ± 3.56%, with 19 patients achieving AUC values above 95%, indicating strong discriminative performance for the majority of patients. Taken together, the segment-level metrics, event-level metrics, and AUC results demonstrate that the proposed method achieved robust detection performance on the CHB-MIT dataset and was able to accommodate, to a certain extent, inter-patient differences in seizure manifestations and EEG background activity.

**Figure 7:**
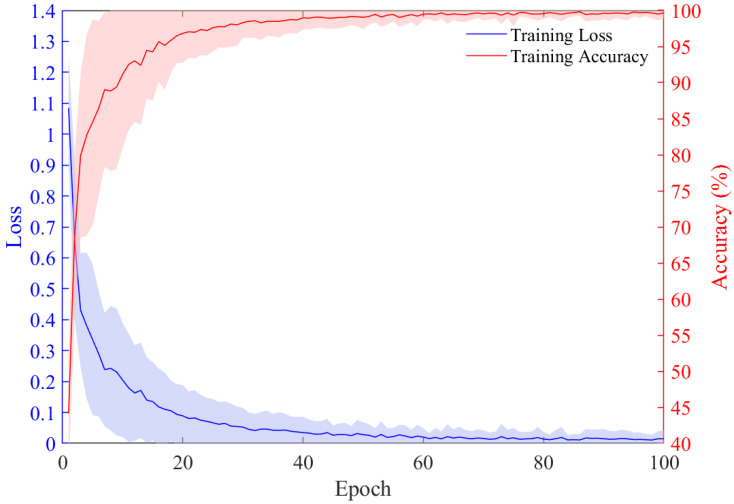
Average cross-entropy training loss and average training accuracy curves for 23 patients in the CHB-MIT dataset. The shaded region indicates the standard deviation.

We further summarized the average cross-entropy training loss curves and average training accuracy curves of the 23 patients in the CHB-MIT dataset, as shown in Figure 7. As the number of training epochs increased, the average training loss continuously decreased and gradually stabilized in the later stage. Meanwhile, the average training accuracy rose rapidly in the early phase of training and remained at a high level after approximately 50 epochs. These observations indicate that the proposed 3D-CNN-ViT model achieved stable parameter optimization and effective feature learning under the current training strategy, and that the overall training process exhibited expected convergence.

Although the model achieved high overall detection performance on the CHB-MIT dataset, noticeable differences remained across patients. As shown in Table 4, patients 6, 13, 14, 20, and 24 exhibited FDR values substantially higher than the overall average, while patients 4, 18, and 24 showed relatively low AUC values. One important reason for these performance fluctuations is that some cases had shorter average seizure durations and more limited seizure durations available for training. For example, the average seizure durations of patients 6, 14, 20, and 24 were only 15.30 s, 21.13 s, 36.75 s, and 31.94 s, respectively, and the corresponding training seizure durations were only 1.07 min, 0.23 min, 0.48 min, and 0.42 min. For such short seizures, 4-s segmentation together with the smoothing, thresholding, and collar expansion used in postprocessing can more easily amplify high-score segments near seizure boundaries, thereby increasing false alarms.

In addition, the CHB-MIT dataset is a pediatric long-term scalp EEG database, and substantial differences often exist among seizures in terms of onset rhythm, spatial distribution, and time–frequency characteristics. In patient-specific detection tasks, model performance is more likely to fluctuate when the test seizures differ in presentation from the training seizures. Moreover, although retaining only the 18 shared bipolar channels ensured consistent input dimensionality, this step may also have weakened patient-specific spatial information for cases with more focal seizure onset or more spatially restricted onset zones. Therefore, the relatively high FDR or relatively low AUC observed in a few patients more likely reflects greater seizure-pattern complexity and insufficient training-sample coverage, rather than complete model failure in those cases.

### 4.2. Results on SH-SDU Database

The detection results on the SH-SDU dataset are presented in Table 5. For the 6 patients included in the final evaluation, the proposed method achieved good overall segment-level performance, with average sensitivity, specificity, and accuracy of 92.05 ± 11.27%, 97.53 ± 1.94%, and 96.37 ± 3.74%, respectively. Segment-level sensitivity reached 100% in 2 patients, and accuracy exceeded 98% in 3 patients, indicating that the method effectively distinguished seizure from non-seizure segments in most cases.

At the event level, the method also exhibited strong detection capability. As shown in Table 5, experts annotated 64 seizure events, of which 60 were successfully detected. The macro-averaged event-based sensitivity across patients was 96.08 ± 9.61%, while the pooled event-level detection ratio was 60/64 = 93.75%. Among the 6 patients, 5 achieved 100% event-based sensitivity, and only 1 patient did not have all seizure events detected. Meanwhile, the average false detection rate (FDR) was 0.69 ± 0.57/h, and 5 patients had an FDR below 1/h, indicating that the method can maintain a high event-detection rate while effectively suppressing false-positive events.

To further evaluate the overall discriminative ability of the model output scores for seizure and non-seizure states, the AUC was also computed for each patient. As shown in Table 5, the average AUC reached 92.89 ± 8.67%; 4 patients had AUC values above 90%, among them 3 patients achieved AUC values above 99%. Taken together, the segment-level metrics, event-level metrics, and AUC results indicate that the proposed method achieved reliable detection performance on the SH-SDU dataset, suggesting that it is applicable not only to public datasets but also to real clinical data.

**Figure 8:**
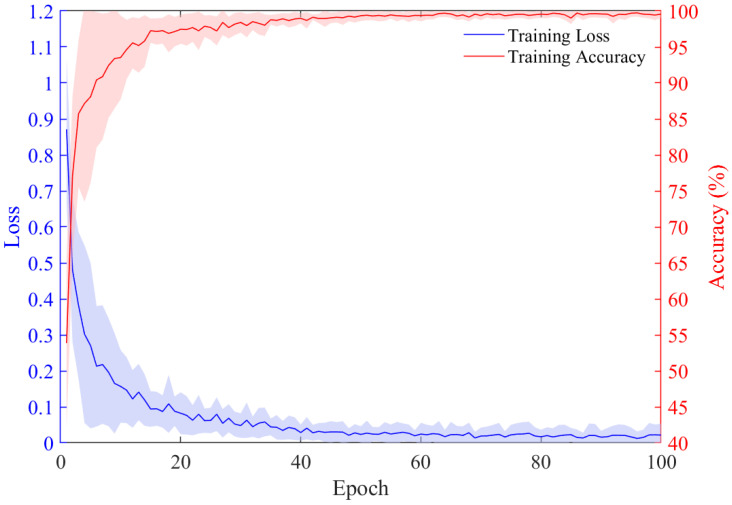
Average cross-entropy training loss and average training accuracy curves for 6 patients in the SH-SDU dataset. The shaded region indicates the standard deviation.

We further summarized the average cross-entropy training loss curves and average training accuracy curves of the 6 patients in the SH-SDU dataset, as shown in Figure 8. As the number of training epochs increased, the average training loss continuously decreased and gradually stabilized in the later stage. Meanwhile, the average training accuracy increased rapidly during early training and remained at a high level in later epochs. Compared with the CHB-MIT dataset, the SH-SDU dataset was collected in a real clinical environment and contains fewer cases with greater inter-subject variability; consequently, the fluctuations in the early training stage were somewhat larger. Even so, the overall convergence trend remained stable. These findings indicate that the proposed 3D-CNN-ViT model also achieved effective parameter optimization and discriminative feature learning on the SH-SDU dataset, demonstrating good training stability.

Although the model achieved reliable overall detection performance on the SH-SDU dataset, inter-patient differences remained pronounced. As shown in Table 5, patient 1 had segment-level sensitivity, event-based sensitivity, and AUC values well below the overall averages, namely 70.98%, 76.47%, and 78.00%, respectively. In addition, this patient had 17 annotated seizure events, of which only 13 were detected, indicating that the seizure patterns in this case were relatively complex and more difficult for the model to distinguish from non-seizure states. By contrast, patients 3, 4, 5, and 6 all achieved 100% event-based detection, although patient 3 still exhibited an FDR of 1.67/h, substantially higher than the overall average, suggesting that the model produced more false alarms while maintaining a high detection rate. Patient 4 also achieved 100% event-based sensitivity, but its AUC was only 88.35%, lower than that of most other cases, indicating more limited separability of its segment-level score distribution. Patient 6, on the other hand, achieved the best performance at both the segment and event levels, with sensitivity, specificity, accuracy, and event-based sensitivity all reaching 100%, an FDR of 0, and an AUC of 99.84%, demonstrating that the proposed method can achieve highly favorable detection performance in some clinical cases.

## 5. Discussion

### 5.1. Ablation Study

On CHB-MIT Patient 12, the ablation study was divided into five stages: Stage A examined the choice of input modality and the fusion strategy; Stage B analyzed the 3D-CNN structure with the ViT configuration fixed; Stage C analyzed the ViT structure with the CNN configuration fixed; Stage D investigated whether locally optimal modules could still improve overall performance after recombination; and Stage E compared the suitability of different time–frequency transforms within the same A00 framework.

#### 5.1.1. Stage A: Modality and Fusion Ablation

Stage A was designed to evaluate the effects of input modality selection and the hybrid architecture. As shown in Table 6, model A00 achieved the most balanced overall performance, with ACC, sensitivity, specificity, event-based sensitivity, and AUC reaching 98.28%, 97.91%, 97.95%, 100.00%, and 99.16%, respectively, while keeping the FDR at 1.11/h. Under the single-modality CNN setting, A02 outperformed A01 in specificity, ACC, AUC, and FDR, suggesting that phase spectrograms provide useful discriminative information for seizure detection in the proposed framework. After introducing the ViT module, A04 improved over A01, whereas A05 did not further improve over A02, indicating that power features benefited more from global dependency modeling while phase features were more effectively captured by local convolutional structures. In contrast, naive concatenation of power and phase inputs without the ViT branch caused a clear performance decline in A03, suggesting that dual-modality features require an appropriate fusion architecture rather than simple concatenation. Therefore, Stage A supports the adoption of the hybrid power-phase CWT with 3D-CNN–ViT design used in this study, consistent with recent findings on joint power-phase modeling and CNN–ViT collaborative design [32].

To make the ablation labels in Stages B and C self-explanatory, the corresponding configuration changes are summarized in Table 7. Unless otherwise specified, only the target component was changed in each ablation experiment, while the remaining modules, training settings, and postprocessing procedure were kept consistent with the baseline model.

#### 5.1.2. Stage B: CNN Ablation with Fixed ViT

With the ViT configuration fixed, Stage B further analyzed the influence of the width, depth, and pooling strategy of the 3D-CNN on the final results. As shown in Table 8, the different CNN variants did not exhibit a simple pattern whereby deeper or wider necessarily led to better performance. Instead, they showed distinct metric-oriented trade-offs. For example, in the width ablation, model B04 achieved the highest ACC in Stage B (98.23%), while also reaching an AUC of 99.09%, indicating that a moderate increase in channel width can improve overall discriminative ability. However, its FDR was 1.74/h, higher than the 1.11/h of model A00, showing that a wider CNN did not simultaneously improve false-alarm control.

In contrast, the weak-pooling configuration B11 achieved the highest AUC in Stage B (99.19%) and the lowest FDR (0.63/h), suggesting that retaining more local detail helps suppress false alarms and improve ranking ability. However, its segment-based sensitivity dropped to 96.23%, lower than the 97.91% of model A00, indicating that its advantage lies mainly in a low-false-alarm operating regime rather than in complete coverage of seizure segments. The deeper configuration model B07 showed a similar tendency, with FDR reduced to 0.97/h, but sensitivity and ACC still failed to surpass model A00. Overall, Stage B indicates that CNN structure substantially changes the trade-off among sensitivity, AUC, and FDR, yet no CNN variant can replace the current baseline across all core metrics.

#### 5.1.3. Stage C: ViT Ablation with Fixed CNN

Stage C directly evaluates the contribution of the ViT module. As shown in Table 9, removing ViT in C01 caused the most obvious degradation: ACC, sensitivity, and specificity decreased to 83.93%, 82.43%, and 82.46%, respectively; AUC fell to 89.10%; and FDR increased to 5.18/h. These results indicate that CNN-based local convolution alone is insufficient to fully exploit the fused power-phase features, and that ViT-based global dependency modeling is an important component of the proposed framework. This observation is consistent with recent hybrid deep models in which convolutional modules capture local features while Transformer-like modules model long-range dependencies [49]. Adjustments to ViT depth, attention heads, and patch size further reveal a complexity-performance trade-off. Although the deeper C05 configuration achieved the highest ACC and AUC among the Stage C variants, it also increased model complexity, and other variants with lower FDR often showed reduced sensitivity. Overall, the baseline configuration used in this study, namely a single-layer ViT with 4 attention heads, 64-dimensional embeddings, and medium-sized patches, provides a favorable balance among performance, complexity, and structural simplicity.

To further clarify the design choice of the ViT branch, we additionally compared the parameter size and detection performance of models using ViT encoders with 0, 1, 2, and 3 layers. As shown in Table 10, removing the ViT module reduced the number of model parameters to only 17.70k, but all major metrics deteriorated substantially, with ACC and AUC decreasing to 83.93% and 89.10%, respectively, and FDR increasing to 5.18/h. After introducing a single-layer ViT encoder, the parameter count increased to 455.50k, while ACC, event-based sensitivity, and AUC improved markedly to 98.28%, 100.00%, and 99.16%, respectively. Although the 2-layer ViT still maintained a relatively low FDR (1.36/h) and improved sensitivity to 99.58%, its ACC and AUC declined to 97.98% and 98.92%, respectively. Its overall performance was therefore comparable to that of the 1-layer ViT, while its 816.7k parameters would substantially increase computational cost and model complexity, offering no clear advantage when performance gains are weighed against lightweight deployment. The 3-layer ViT further increased model complexity to 1.1M parameters, yet instead caused decreases in segment-level sensitivity and ACC. Thus, from the combined perspective of effectiveness, compactness, and structural simplicity, the single-layer ViT adopted in the baseline remains the most reasonable choice.

To further examine the combined influence of ViT encoder depth and L2 regularization, an additional controlled experiment was conducted on CHB-MIT Patient 12. The ViT encoder depth was set to 1, 2, and 3, and the L2 regularization coefficient was varied among 0, $10^{-3}$, $10^{-2}$, and $10^{-1}$. All other training settings and data partitions were kept unchanged. Evaluation was performed on a balanced unseen testing subset consisting of 275 seizure samples and 275 non-seizure samples. As shown in Table 11, the model with a single-layer ViT encoder and an L2 regularization coefficient of $10^{-3}$ achieved the highest accuracy of 91.27%. Therefore, this setting was adopted in the proposed framework.

#### 5.1.4. Stage D: Joint Verification

Stage D was designed to determine whether CNN or ViT modules that performed well individually would still improve overall performance after recombination. As shown in Table 12, locally optimal modules could not be directly converted into a globally optimal model through simple replacement. For example, in model D02, combining a better-performing CNN module with the baseline ViT produced an FDR of 1.11/h, essentially the same as that of model A00, but neither ACC nor AUC exceeded those of model A00. This suggests that the variant may be better interpreted as a low-false-alarm candidate rather than as a direct replacement for the final main model.

Furthermore, when the low-false-alarm CNN was recombined with a stronger ViT in model D04, performance did not improve further. Instead, FDR increased to 3.00/h and sensitivity decreased to 92.05%. This indicates that model performance is not determined by a simple addition of locally optimal modules, but rather by the overall matching relationship among the input modality, the local modeling ability of the CNN, and the global modeling ability of the ViT.

#### 5.1.5. Stage E: Time–Frequency Transform Ablation

In addition to ablating the network structure, we compared different time–frequency transforms under the same A00 architecture, while keeping the training strategy and postprocessing unchanged. As shown in Table 13, the S-transform showed the weakest performance, whereas WSST maintained 100.00% event-based sensitivity but produced a higher FDR of 4.07/h. Among the alternative transforms, STFT achieved the best overall performance, with ACC, specificity, and AUC values of 98.13%, 97.92%, and 99.06%, respectively. Nevertheless, compared with the final CWT-based model, STFT still yielded slightly lower segment-based sensitivity, specificity, ACC, and AUC, together with a slightly higher FDR. These results indicate that the choice of time–frequency transform affects final detection performance and that CWT remains the more appropriate choice for the current power-phase 3D-CNN–ViT framework [50].

### 5.2. Visualization and Interpretability Analysis

#### 5.2.1. t-SNE Visualization Analysis

**Figure 9:**
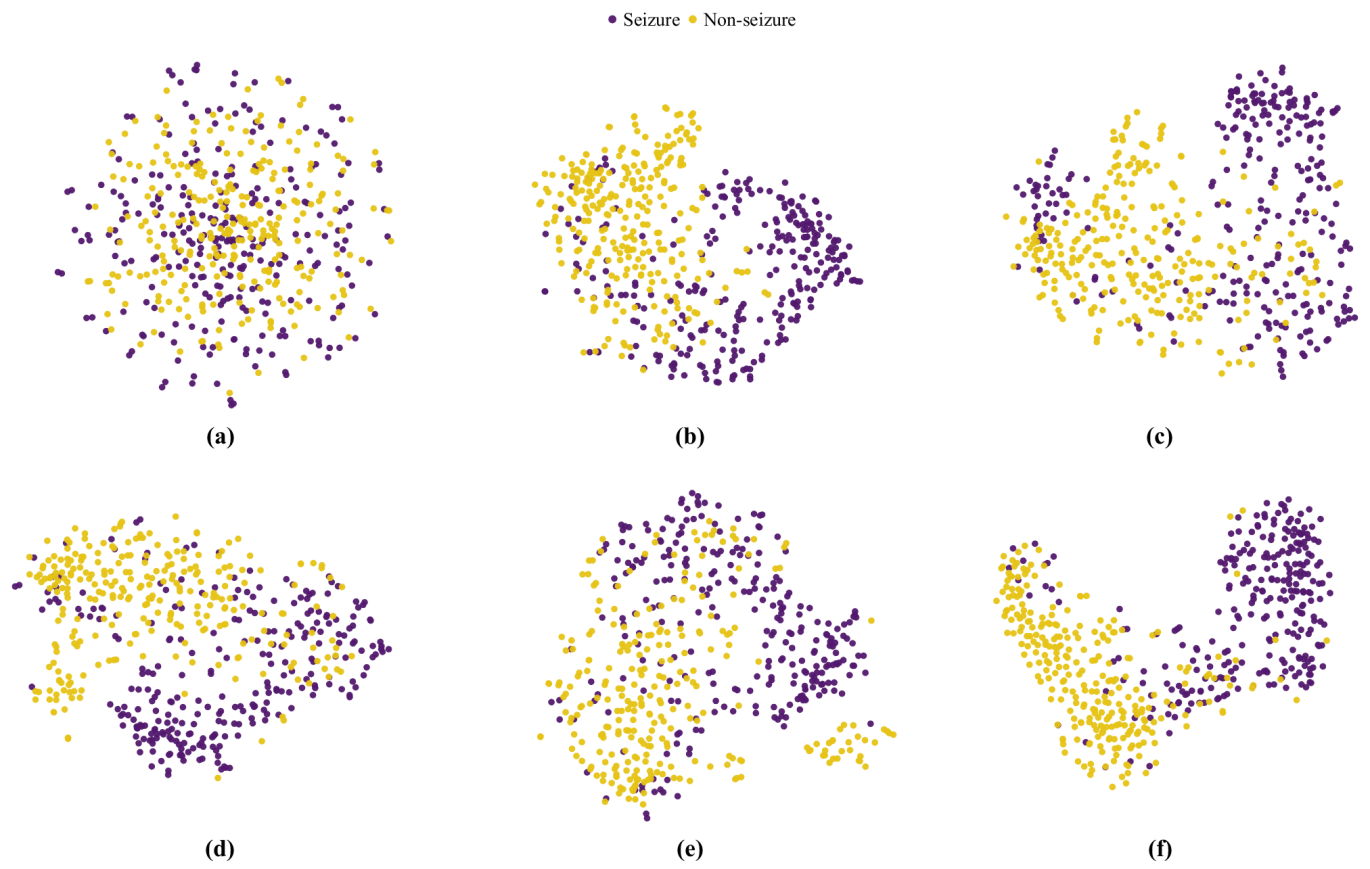
t-SNE visualization of feature representations at different stages of the proposed model using unseen testing samples from CHB-MIT Patient 12. A balanced subset containing all 275 seizure testing samples and 275 randomly selected non-seizure testing samples was used. (**a**) Original EEG. (**b**) Fused CWT input. (**c**) Feature representation after the first 3D max-pooling layer. (**d**) Feature representation after the second 3D max-pooling layer. (**e**) Deep 3D-CNN feature representation. (**f**) Final ViT feature representation. The final feature space shows clearer separation between seizure and non-seizure testing samples.

To better evaluate feature generalization on unseen data, the t-SNE visualization was regenerated using testing samples from CHB-MIT Patient 12 that were not used during model training. A balanced subset containing all 275 seizure testing samples and 275 randomly selected non-seizure testing samples was used only for visualization to reduce the visual bias caused by class imbalance. As a classic nonlinear dimensionality reduction method, t-SNE is widely used to visualize separability of deep features [51]. As shown in Figure 9, the original EEG and fused CWT input representations still exhibited considerable overlap between seizure and non-seizure samples, whereas the learned features became progressively more separable after the 3D-CNN blocks. The final ViT feature representation showed the clearest separation, suggesting that the proposed hybrid 3D-CNN–ViT architecture learned discriminative feature representations that remained separable on unseen testing data.

#### 5.2.2. Grad-CAM-Based Interpretability Analysis

**Figure 10:**
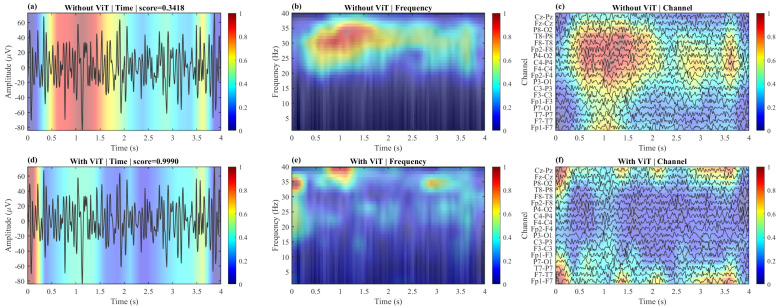
Grad-CAM visualization of a representative corrected false-negative seizure segment from Patient 12 in the CHB-MIT dataset. The seizure class was used as the target class. (**a**–**c**) Temporal, frequency–time, and channel–time Grad-CAM responses of the 3D-CNN-only model without the ViT branch, respectively. (**d**–**f**) Corresponding responses of the full 3D-CNN-ViT model. For both models, Grad-CAM was computed with respect to the last 3D-CNN activation feature map before patch embedding. In the full model, gradients were further backpropagated from the final seizure-class score through the ViT encoder and patch embedding module to this feature map. Warmer colors indicate larger positive contributions to the seizure-class decision.

To further examine how the ViT branch influences the decision process of the proposed model, we performed a Grad-CAM-based interpretability analysis on the CHB-MIT dataset. Grad-CAM generates class-discriminative response maps by using the gradient information of the target class with respect to intermediate feature maps, thereby providing a qualitative indication of the regions that contribute to the model decision [52]. In this analysis, the seizure class was used as the target class. Grad-CAM was used to compare the 3D-CNN-only model without the ViT branch and the full 3D-CNN-ViT model. For both models, Grad-CAM was computed with respect to the last 3D-CNN activation feature map before patch embedding. Transformer token embeddings and attention matrices were not used as the target maps for Grad-CAM. In the 3D-CNN-only model, gradients were backpropagated from the final seizure-class score of the CNN classifier to this feature map. In the full 3D-CNN-ViT model, gradients were backpropagated from the final seizure-class score through the ViT encoder and patch embedding module to the same 3D-CNN feature map. Therefore, the responses in Figure 10 compare the class-discriminative regions used by the convolutional model alone and by the full model after ViT-based global dependency modeling. For visualization, the Grad-CAM response was resized to the input power-phase feature size and summarized as temporal, frequency–time, and channel–time responses.

We selected a representative seizure segment from Patient 12 for visualization. This segment was falsely classified as a non-seizure segment by the CNN-only model but was correctly detected as a seizure segment after introducing the ViT branch. We refer to this type of sample as a corrected false-negative case (fix-FN), indicating that the false-negative prediction of the CNN-only model was corrected by the full 3D-CNN-ViT model. The corresponding Grad-CAM results are shown in Figure 10. For the CNN-only model, the output seizure score of this segment was 0.3418, indicating that the model failed to assign a sufficiently high seizure probability. Its Grad-CAM responses were mainly concentrated in several local regions. In the temporal view, relatively high responses appeared within a limited time interval, while later portions of the segment contributed less to the decision. After introducing the ViT branch, the seizure score increased to 0.9990, and the segment was correctly detected as a seizure segment. The corresponding Grad-CAM maps show that the response distribution was substantially changed. In the temporal view, the high-response regions became less confined to the initial local interval and were redistributed across the segment. These visualizations provide qualitative evidence that the ViT branch changes the class-discriminative response pattern of the model.

### 5.3. Computational Efficiency

To evaluate the computational efficiency of the proposed framework, preprocessing time, patient-specific training time, and inference speed were measured on all 23 CHB-MIT patients included in the final evaluation. As shown in Table 14, the average preprocessing time was 0.057 ± 0.026 s per 4-s EEG segment, the average patient-specific training time was 18.26 ± 11.54 s, and the average inference speed reached 191.13 ± 37.63 segments/s. Since patient-specific training is performed offline, the main online computational cost during continuous monitoring comes from preprocessing and inference. The measured preprocessing time and inference speed indicate that the proposed framework has acceptable computational efficiency for continuous EEG seizure detection.

### 5.4. Benchmarking Against Recent CHB-MIT Studies

To further position the proposed method relative to recent related studies, Table 15 summarizes nine representative seizure detection studies based on the CHB-MIT dataset published since 2021 and compares them with the results of the present study. The table lists the feature extraction methods, classifiers, and both segment-level and event-level evaluation metrics.

As shown in Table 15, the proposed method achieved a sensitivity of 98.68%, a specificity of 98.33%, an accuracy of 98.49%, an event-based sensitivity of 99.13%, and an FDR of 0.88/h on the 23 CHB-MIT patients included in the final evaluation. Compared with recent raw-EEG, time–frequency, and hybrid deep-learning methods, the proposed CWT-based power-phase 3D-CNN–ViT framework showed competitive overall performance, particularly in segment-level sensitivity, accuracy, and event-based sensitivity.

To assess cross-patient statistical significance, two-sided paired *t*-tests were conducted using matched patient-wise CHB-MIT results. Compared with Zhong et al. [18], the proposed method achieved significantly higher segment-level sensitivity (98.68 ± 2.97% vs. 95.94 ± 3.49%, p=0.0037) and accuracy (98.49 ± 2.10% vs. 95.98 ± 3.57%, p=0.0077). In comparison with the CWT-based dual-branch model proposed by Wang et al. [32], after excluding chb16 to match the 23 patients used in this study, the proposed method showed higher and comparable segment-level sensitivity (98.68 ± 2.97% vs. 98.00 ± 4.59%, p=0.5474), specificity (98.33 ± 2.31% vs. 98.14 ± 4.11%, p=0.8457), accuracy (98.49 ± 2.10% vs. 98.39 ± 2.89%, p=0.8958), and event-based sensitivity (99.13 ± 4.17% vs. 98.90 ± 4.26%, p=0.3282). Nevertheless, our model has only one branch, which is more efficient than a dual-branch network architecture. In addition, compared with the mutual patients reported in the DWT-based work of Cao et al. [53], the proposed method showed slightly higher sensitivity (98.95 ± 2.72% vs. 98.12 ± 1.09%, p=0.1769), while its specificity (98.41 ± 2.34% vs. 99.33 ± 0.56%, p=0.0759) and accuracy (98.61 ± 2.07% vs. 98.66 ± 0.70%, p=0.9101) were slightly lower. More importantly, their CNN-BiLSTM framework involves high computational complexity and a large number of model parameters, whereas the proposed model uses only three 3D-CNN layers and a single ViT encoder layer, with approximately 455.5k learnable parameters.

Although CHB-MIT and SH-SDU were originally recorded with different sampling rates and montage configurations, the proposed framework achieved stable detection performance on both datasets after unified preprocessing. Specifically, CHB-MIT mainly uses bipolar derivations, whereas SH-SDU was recorded using a monopolar montage. Bipolar and monopolar montages may lead to different amplitude distributions, spatial emphasis, and inter-channel relationships in EEG signals. However, they are different derivations used in clinical EEG interpretation, and seizure-related waveform patterns can remain comparable across montages when similar electrode locations are used [54,55]. Therefore, the results on both datasets indicate that the proposed power-phase representation based on CWT and hybrid 3D-CNN–ViT model is applicable to both bipolar and monopolar EEG recordings under the current patient-specific evaluation setting.

**Table 15:** Comparison with nine representative CHB-MIT seizure-detection studies published since 2021.

Author	Year	FeatureExtraction	Classifier	Sensitivity	Specificity	Accuracy	Event-BasedSensitivity	FDR(/h)
Wang et al. [56]	2021	Raw EEG	Stacked 1D-CNN	88.14%	99.62%	99.54%	99.31%	0.20
Zhong et al. [18]	2024	Stockwell transform	Transformer	96.11%	96.38%	96.15%	96.57%	0.38
Shen et al. [57]	2024	STFT spectrograms	GoogleNet CNN	98.90%	—	97.74%	—	—
Dong et al. [58]	2024	Raw EEG	TCN-BiLSTM	94.31%	97.13%	97.09%	96.48%	0.38
Cui et al. [59]	2024	DWT-filtered raw EEG	CNN-Reformer	97.57%	98.11%	98.09%	96.81%	0.27
Cao et al. [53]	2025	DWT + hand-crafted time–frequency	CNN-Bi-LSTM	97.84%	99.21%	98.43%	—	—
Li et al. [49]	2025	DWT-filtered raw EEG	CNN-Informer	99.54%	98.55%	98.54%	99.07%	0.16
Liu et al. [60]	2025	DWT-filtered raw EEG	Group CosCNN	97.70%	97.54%	—	—	—
Wang et al. [32]	2025	CWT power + phase spectrograms	Dual-branch CNN-ViT	98.09%	98.21%	98.45%	98.95%	0.31
This work	2026	CWT power + phase spectrograms	3D-CNN + ViT	98.68%	98.33%	98.49%	99.13%	0.88

## 6. Conclusions

To address the limited feature utilization and insufficient global information extraction of conventional methods, we proposed a seizure detection framework that integrates CWT-based time–frequency transformation with a hybrid 3D-CNN-ViT network. By constructing a fused power-phase volume for each EEG segment and using the 3D-CNN to extract local discriminative features while the ViT models higher-level global associations, the framework achieves effective identification of seizure segments. Experimental results on the CHB-MIT and SH-SDU datasets show that the proposed model achieved AUC values of 97.26% and 92.89%, segment-level sensitivities of 98.68% and 92.05%, specificities of 98.33% and 97.53%, and accuracies of 98.49% and 96.37%, respectively. In event-level evaluation, the model achieved sensitivities of 99.13% and 96.08%, with corresponding false detection rates of 0.88/h and 0.69/h. These findings demonstrate that the proposed method not only delivers strong detection performance on a public benchmark dataset but also exhibits good applicability and stability on real clinical data. From a clinical application perspective, the proposed framework should be regarded as an auxiliary tool for identifying candidate ictal EEG segments in long-term EEG monitoring rather than a replacement for expert clinical diagnosis or semeiological assessment. Future work will further expand validation using larger multicenter clinical EEG datasets, incorporate clinical semiology information related to the preictal, ictal, and postictal phases, investigate cross-patient and patient-independent modeling strategies, and optimize model lightweighting and real-time deployment, thereby enhancing the practical utility of the system for long-term clinical monitoring.

## Figures and Tables

**Table 1:** Summary of the CHB-MIT dataset used in this study.

Patient	Sex	Age	Total Duration (h)	Number of Used Seizures	Mean Seizure Duration (s)	Training Seizure Duration (min)	Training Non-Seizure Duration (min)
1	F	11	40.55	7	63.15	0.67	3.33
2	M	11	35.27	3	57.34	1.35	6.75
3	F	14	38.00	7	57.43	0.87	4.33
4	M	22	156.07	4	94.50	0.82	4.08
5	F	7	39.00	5	111.60	1.92	9.58
6	F	1.5	66.74	10	15.30	1.07	5.33
7	F	14.5	67.05	3	108.34	1.43	7.17
8	M	3.5	20.01	5	183.80	2.85	14.25
9	F	10	67.87	4	69.00	1.07	5.33
10	M	3	50.02	6	65.50	0.58	2.92
11	F	12	34.79	3	268.67	0.37	1.83
12	F	2	20.69	27	36.63	2.15	10.75
13	F	3	33.00	12	44.59	3.48	17.42
14	F	9	26.00	8	21.13	0.23	1.17
15	M	16	40.01	20	99.60	2.08	10.42
17	F	12	21.01	3	97.67	1.50	7.50
18	F	18	35.63	6	52.84	0.83	4.17
19	F	19	29.93	3	78.67	1.30	6.50
20	F	6	27.60	8	36.75	0.48	2.42
21	F	13	32.83	4	49.75	0.93	4.67
22	F	9	31.00	3	68.00	0.97	4.83
23	F	6	26.56	7	60.58	1.88	9.42
24	–	–	21.30	16	31.94	0.42	2.08
Summary	–	–	960.93	174	–	29.25	146.27

Note: Patient chb16 was excluded because most of their seizure events were extremely short.

**Table 2:** Summary of the SH-SDU dataset used in this study.

Patient	Sex	Age	Total Duration (h)	Number of Used Seizures	Mean Seizure Duration (s)	Training Seizure Duration (min)	Training Non-Seizure Duration (min)
1	F	28	20.58	19	40.53	0.95	4.75
2	M	61	16.04	10	220.80	5.78	28.92
3	M	34	12.00	10	52.20	1.57	7.83
4	M	72	15.56	29	109.38	3.18	15.92
5	M	76	18.00	5	240.60	2.28	11.42
6	F	38	6.00	3	34.67	0.53	2.67
Summary	–	–	88.18	76	–	14.30	71.50

**Table 3:** Detailed configuration of the 3D-CNN feature extractor.

Layer	Kernel/Pool	Stride	Padding	Output Size	Operation
Input	–	–	–	40×128×36×1	Fused input
Conv3D-1	3×3×3	1×1×1	same	40×128×36×8	Conv3D+BN+ReLU
MaxPool3D-1	2×4×2	2×4×2	same	20×32×18×8	Max pooling
Conv3D-2	3×3×3	1×1×1	same	20×32×18×16	Conv3D+BN+ReLU
MaxPool3D-2	2×2×2	2×2×2	same	10×16×9×16	Max pooling
Dropout	–	–	–	10×16×9×16	Dropout, p=0.2
Conv3D-3	3×3×3	1×1×1	same	10×16×9×32	Conv3D+BN+ReLU

**Table 4:** Results of the proposed method on the CHB-MIT dataset.

Patient No.	Segment-Based Evaluation Results			Event-Based Evaluation Results				AUC
	Sensitivity	Specificity	Accuracy	$N_{e}$	$N_{d}$	Sensitivity	FDR (/h)	
1	100.00%	99.88%	99.92%	6	6	100.00%	0.02	99.93%
2	100.00%	98.81%	99.21%	2	2	100.00%	1.33	99.42%
3	100.00%	98.55%	99.03%	6	6	100.00%	0.34	99.12%
4	98.84%	99.31%	99.35%	3	3	100.00%	0.12	86.13%
5	100.00%	99.87%	99.89%	4	4	100.00%	0.03	94.60%
6	100.00%	96.51%	97.68%	6	6	100.00%	4.87	98.25%
7	100.00%	99.43%	99.62%	2	2	100.00%	0.10	98.72%
8	100.00%	99.25%	99.50%	4	4	100.00%	0.25	99.49%
9	100.00%	99.90%	99.93%	3	3	100.00%	0.04	97.85%
10	100.00%	99.81%	99.87%	5	5	100.00%	0.02	97.11%
11	100.00%	99.90%	99.93%	2	2	100.00%	0.03	99.95%
12	97.91%	97.95%	98.28%	23	23	100.00%	1.11	99.16%
13	96.67%	91.59%	93.84%	8	8	100.00%	3.28	96.86%
14	97.83%	97.02%	97.65%	7	7	100.00%	2.38	98.92%
15	99.59%	99.13%	99.35%	19	19	100.00%	0.18	97.42%
17	100.00%	98.98%	99.32%	2	2	100.00%	0.24	97.08%
18	87.50%	97.89%	93.18%	5	4	80.00%	0.34	88.79%
19	100.00%	99.59%	99.69%	2	2	100.00%	0.07	99.36%
20	98.63%	92.05%	94.48%	7	7	100.00%	3.48	95.73%
21	100.00%	99.76%	99.84%	3	3	100.00%	0.06	99.65%
22	100.00%	99.98%	99.99%	2	2	100.00%	0.00	99.97%
23	100.00%	99.79%	99.86%	6	6	100.00%	0.11	99.83%
24	92.65%	96.66%	95.81%	15	15	100.00%	1.83	93.75%
Average	98.68 ± 2.97%	98.33 ± 2.31%	98.49 ± 2.10%	142	141	99.13 ± 4.17%	0.88 ± 1.37	97.26 ± 3.56%

$N_{e}$: Number of seizures marked by experts. $N_{d}$: Number of detected seizures. Values in the Average row are reported as mean ± standard deviation across patients.

**Table 5:** Results of the proposed method on the SH-SDU dataset.

Patient No.	Segment-Based Evaluation Results			Event-Based Evaluation Results				AUC
	Sensitivity	Specificity	Accuracy	$N_{e}$	$N_{d}$	Sensitivity	FDR (/h)	
1	70.98%	98.07%	89.95%	17	13	76.47%	0.97	78.00%
2	88.58%	94.47%	94.41%	8	8	100.00%	0.63	92.36%
3	99.11%	98.33%	98.74%	8	8	100.00%	1.67	99.47%
4	93.61%	96.10%	96.34%	27	27	100.00%	0.45	88.35%
5	100.00%	98.19%	98.80%	2	2	100.00%	0.42	99.33%
6	100.00%	100.00%	100.00%	2	2	100.00%	0.00	99.84%
Average	92.05 ± 11.27%	97.53 ± 1.94%	96.37 ± 3.74%	64	60	96.08 ± 9.61%	0.69 ± 0.57	92.89 ± 8.67%

$N_{e}$: Number of seizures marked by experts. $N_{d}$: Number of detected seizures. Values in the Average row are reported as mean ± standard deviation across patients.

**Table 6:** Ablation results of the input modality and fusion strategy in Stage A.

Model No.	Input	CNN	ViT	Segment-Based Evaluation Results			Event-Based Evaluation Results		AUC
				Sensitivity	Specificity	ACC	Sensitivity	FDR (/h)	
A00	Power + Phase	baseline	baseline	97.91%	97.95%	98.28%	100.00%	1.11	99.16%
A01	Power	baseline	removed	94.56%	84.11%	88.50%	100.00%	4.41	92.41%
A02	Phase	baseline	removed	87.87%	96.33%	95.53%	100.00%	1.40	96.70%
A03	Power + Phase	baseline	removed	76.99%	85.19%	84.12%	86.96%	4.26	89.31%
A04	Power	baseline	baseline	92.47%	96.02%	96.09%	100.00%	3.87	98.06%
A05	Phase	baseline	baseline	80.34%	95.58%	93.78%	100.00%	4.84	94.77%

**Table 7:** Configuration definitions for the ablation labels in Stages B and C.

Stage	Label	Changed Setting	Other Settings
Baseline	A00	CNN: 8–16–32; pool1 = [2,4,2], pool2 = [2,2,2]; ViT: depth = 1, heads = 4, embedDim = 64, patch = [2,2,9]	–
B	B01/Width-S	CNN widths: 2–4–8	ViT fixed
B	B02/Width-M	CNN widths: 4–8–16	ViT fixed
B	B04/Width-L	CNN widths: 16–32–64	ViT fixed
B	B05/Depth-2	CNN depth: 2 blocks, 8–16	ViT fixed
B	B07/Depth-4	CNN depth: 4 blocks, 8–16–32–64	ViT fixed
B	B11/Pool-Weak	pool1 = [2,2,2], pool2 = [2,2,1]	ViT fixed
B	B13/Pool-Strong	pool1 = [2,4,2], pool2 = [2,4,2]	ViT fixed
C	C01/ViT removed	Remove ViT encoder	CNN fixed
C	C03/Depth = 2	ViT depth = 2	CNN fixed
C	C04/Depth = 3	ViT depth = 3	CNN fixed
C	C05/Depth = 4	ViT depth = 4	CNN fixed
C	C06/Heads = 2	Attention heads = 2	CNN fixed
C	C08/Heads = 8	Attention heads = 8	CNN fixed
C	C09/embedDim = 32	Embedding dimension = 32	CNN fixed
C	C11/embedDim = 128	Embedding dimension = 128	CNN fixed
C	C15/Small patch	Patch size = [2,1,9]	CNN fixed
C	C17/Large patch	Patch size = [2,4,9]	CNN fixed

**Table 8:** Ablation results of the 3D-CNN branch in Stage B.

Model No.	Main Change	Segment-Based Evaluation Results			Event-Based Evaluation Results		AUC
		Sensitivity	Specificity	ACC	Sensitivity	FDR (/h)	
B01	Width-S	99.16%	96.15%	97.29%	100.00%	2.18	98.85%
B02	Width-M	97.91%	96.57%	97.37%	100.00%	1.21	98.83%
B04	Width-L	97.91%	97.87%	98.23%	100.00%	1.74	99.09%
B05	Depth-2	96.65%	97.13%	97.53%	100.00%	1.21	98.93%
B07	Depth-4	99.16%	97.20%	97.99%	100.00%	0.97	99.02%
B11	Pool-Strength-Weak	96.23%	97.95%	98.01%	100.00%	0.63	99.19%
B13	Pool-Strength-Strong	97.91%	96.93%	97.60%	100.00%	1.50	98.88%

**Table 9:** Ablation results of the ViT branch in Stage C.

Model No.	Main Change	Segment-Based Evaluation Results			Event-Based Evaluation Results		AUC
		Sensitivity	Specificity	ACC	Sensitivity	FDR (/h)	
C01	ViT removed	82.43%	82.46%	83.93%	91.30%	5.18	89.10%
C03	Depth = 2	99.58%	97.07%	97.98%	100.00%	1.36	98.92%
C04	Depth = 3	94.98%	97.01%	97.17%	100.00%	1.79	98.55%
C05	Depth = 4	99.58%	97.93%	98.55%	100.00%	0.82	99.15%
C06	Heads = 2	98.33%	91.81%	94.26%	100.00%	4.21	96.82%
C08	Heads = 8	92.89%	97.70%	97.28%	100.00%	0.97	98.90%
C09	embedDim = 32	96.23%	97.65%	97.81%	100.00%	1.16	99.04%
C11	embedDim = 128	98.75%	97.01%	97.80%	100.00%	1.16	99.10%
C15	Small patch	98.33%	94.87%	96.30%	100.00%	2.18	96.88%
C17	Large patch	97.49%	97.87%	98.16%	100.00%	0.58	99.01%

**Table 10:** Comparison of parameter size and performance under different ViT depths.

ViT Depth	Parameters	Segment-Based Evaluation Results			Event-Based Evaluation Results		AUC
		Sensitivity	Specificity	ACC	Sensitivity	FDR (/h)	
0 layer	17.7k	82.43%	82.46%	83.93%	91.30%	5.18	89.10%
1 layer	455.5k	97.91%	97.95%	98.28%	100.00%	1.11	99.16%
2 layers	816.7k	99.58%	97.07%	97.98%	100.00%	1.36	98.92%
3 layers	1.1M	94.98%	97.01%	97.17%	100.00%	1.79	98.55%

**Table 11:** Effect of L2 regularization on different ViT encoder depths using a balanced unseen testing subset from CHB-MIT Patient 12.

ViT Depth	L2 Regularization	Accuracy (%)
1	0	90.91
1	$10^{-3}$	91.27
1	$10^{-2}$	86.73
1	$10^{-1}$	89.45
2	0	88.00
2	$10^{-3}$	87.27
2	$10^{-2}$	87.27
2	$10^{-1}$	83.82
3	0	89.27
3	$10^{-3}$	87.27
3	$10^{-2}$	87.64
3	$10^{-1}$	83.82

**Table 12:** Joint validation results in Stage D.

Model No.	CNN Part	ViT Part	Segment-Based Evaluation Results			Event-Based Evaluation Results		AUC
			Sensitivity	Specificity	ACC	Sensitivity	FDR (/h)	
A00	baseline	baseline	97.91%	97.95%	98.28%	100.00%	1.11	99.16%
D02	B04	baseline	97.49%	97.23%	97.73%	100.00%	1.11	98.85%
D04	B11	best ViT	92.05%	97.39%	96.93%	100.00%	3.00	98.73%

**Table 13:** Ablation results of different time–frequency transforms in Stage E.

Model No.	Segment-Based Evaluation Results			Event-Based Evaluation Results		AUC
	Sensitivity	Specificity	ACC	Sensitivity	FDR (/h)	
CWT	97.91%	97.95%	98.28%	100.00%	1.11	99.16%
S-transform	77.82%	90.44%	86.31%	78.26%	2.13	85.93%
STFT	97.07%	97.92%	98.13%	100.00%	1.36	99.06%
WSST	92.47%	94.51%	95.08%	100.00%	4.07	97.91%

**Table 14:** Computational efficiency of the proposed framework measured on all 23 CHB-MIT patients included in the final evaluation.

Metric	Mean ± SD	Range
Preprocessing time per 4-s segment	0.057 ± 0.026 s	0.046–0.141 s
Training time per patient	18.26 ± 11.54 s	5.31–53.79 s
Inference speed	191.13 ± 37.63 segments/s	119.98–308.92 segments/s

## Data Availability

The data are available from the corresponding author upon reasonable request, subject to ethical and privacy restrictions.

## Abbreviations

EEG	Electroencephalography
CHB-MIT	Children’s Hospital Boston–Massachusetts Institute of Technology
SH-SDU	Second Hospital of Shandong University
CWT	Continuous wavelet transform
DWT	Discrete wavelet transform
CNN	Convolutional neural network
3D-CNN	Three-dimensional convolutional neural network
ViT	Vision Transformer
BN	Batch normalization
ReLU	Rectified linear unit
MAF	Moving-average filtering
ROC	Receiver operating characteristic
AUC	Area under the curve
FDR	False detection rate
TP	True positive
TN	True negative
FP	False positive
FN	False negative
